# Whole-genome sequencing and comparative genomics reveal the potential pathogenic mechanism of *Neoscytalidium dimidiatum* on pitaya

**DOI:** 10.1128/spectrum.02733-23

**Published:** 2023-11-21

**Authors:** Meng Wang, Min Xu, Zhouwen Wang, Yi Ding, Shaoling Kang, Senrong Jiang, Shuangshuang Wei, Jun Xie, Jiaquan Huang, Dongdong Li, Wenbin Hu, Hongli Li, Xingyu Jiang, Hua Tang

**Affiliations:** 1 School of Breeding and Multiplication, Hainan University, Sanya, China; 2 School of Tropical Agriculture and Forestry, Hainan University, Haikou, China; 3 College of Life Sciences, China Jiliang University, Hangzhou, China; 4 College of Life Sciences, Hainan University, Haikou, China; 5 Tropical Crops Genetic Resources Institute, Chinese Academy of Tropical Agricultural Sciences, Haikou, China; 6 National Center of Technology Innovation for Saline-Alkali Tolerant Rice/College of Coastal Agricultural Sciences, Guangdong Ocean University, Zhanjiang, China; Beijing Forestry University, Beijing, China

**Keywords:** *Neoscytalidium dimidiatum*, genome sequencing, comparative genome analysis, pitaya canker

## Abstract

*Neoscytalidium dimidiatum* (class *Dothideomycetes*) is a fungus responsible for canker disease in pitaya stems and fruits, leading to significant economic losses. However, little is known about the pathogenesis, family evolution, and genetic variants of this species. In this study, we report a high-quality genome sequence of *N. dimidiatum* based on the Nanopore sequencing technology platform for sequencing and Hi-C assembly technology for genome assembly. The genome contains 12 chromosomes (2*n* = 2× = 12; diploid), with a sequencing depth of 186.1×, encoding 12,349 proteins. Molecular phylogenetic analysis showed that *N. dimidiatum* is evolutionarily close to *Botryosphaeria dothidea*. Compared to other fungi, the *N. dimidiatum* genome contains many carbohydrate-active enzymes and secondary metabolites. Additionally, we predict that *N. dimidiatum* contains 121 candidate effectors that may play important roles in infection and colonization, promoting pathogenicity in pitaya. Nine of these effectors were confirmed to contain signal peptides and inhibit BAX/INF1-induced necrosis in *Nicotiana benthamiana*, demonstrating their importance during infection. Finally, we also confirm that *N. dimidiatum* does not form an appressorium or infection thread but instead infects pitaya via open stomata. In conclusion, the results provide a foundation for future research on *N. dimidiatum* and the control of pitaya canker.

**IMPORTANCE:**

Pitaya canker is a significant disease in the pitaya industry in China, causing significant economic losses. Therefore, systematic research on *Neoscytalidium dimidiatum*, the fungus implicated in pitaya canker, is essential for comprehending the pathogenesis of this disease and developing effective control strategies. We applied comparative genomics to reveal the genetic evolution, metabolic diversity, environmental adaptation, and pathogenicity of *N. dimidiatum*, providing ideal targets for studies of pathogenesis and molecular targets for fungicide development. Moreover, the systematic study of the *N. dimidiatum* growth cycle, morphological characteristics, and molecular phylogenetic analysis can promote a comprehensive understanding of its genetic basis.

## INTRODUCTION

Pitaya is a perennial fruit crop belonging to the *Cactaceae* that originated in Latin America (1 – 3). It is now widely cultivated in Asian countries, including China, Malaysia, Vietnam, and Thailand (3, 4). The pitaya fruit is an excellent source of vitamins, dietary fiber, amino acids, and sugars, making it a globally important fruit crop (1, 4).

Pitaya is adapted to hot, dry climates, poor soils, and disease pressure (5). However, one of the most significant problems faced by pitaya cultivators in China is pitaya canker, which is caused by the semi-biotrophic fungal pathogen *Neoscytalidium dimidiatum* (5). This pathogen is highly prevalent in pitaya plantations and is the most severe threat to pitaya growth (5, 6), resulting in an annual loss of approximately 27 million USD in China. In recent years, *N. dimidiatum* has been isolated in many regions, particularly in China’s pitaya cultivation areas (6, 7).

*Neoscytalidium dimidiatum* belongs to the class *Dothideomycetes* and the *Botryosphaeriaceae* family, known for being destructive blight and canker pathogens of plants (8, 9). Members of this family can establish infections through wounds or natural openings such as lenticels and stomata (9). They secrete plant cell wall-degrading enzymes (PCWDEs), toxins, and other effector proteins that invade plants and suppress disease resistance gene expression (10, 11). Lignin peroxidase, laccase, and polyphenol oxidase are released to help them to access nutrients and protect against host defenses (11, 12). For example, *Neofusicoccum parvum* and *Diplodia seriata* encode many PCWDEs, particularly pectate lyases, which cause local degradation of the plant cell wall, allowing the fungi to enter the cells to take up nutrients in the plant and disrupting the plant cell structure (9, 10, 13). Additionally, *Fusarium graminearum*, *Lasiodiplodia theobromae*, and *Blumeria graminis*, secrete large amounts of lipase (10, 14, 15), which is crucial for fungal penetration of host tissues (16), growth and adhesion, and manipulation of host defenses (17). Numerous studies have reported that the effectors of phytopathogenic play a crucial role in plant invasion, with *Cercospora sojina* being screened for 233 effectors, of which the majority were found to be virulence factors and demonstrated the ability to suppress immune responses in soybean (18). Similarly, *Ustilaginoidea* virens was analyzed for 119 effectors, with 13 being shown to trigger death in *N. benthamiana* (19). Among these, the *SCRE2* effector gene was identified as able to impair the host immune response (19). In contrast, the number of carbohydrases and effectors was significantly reduced in *Colletotrichum gloeosporioides*, an ectophytic fungus that avoided causing host death (20).

Despite recent screening for canker-resistant varieties of pitaya being unsuccessful, commercially available broad-spectrum fungicides have been successful, though there can be poor efficiency and increased resistance. In addition to infecting pitaya, *N. dimidiatum* has been isolated and identified on vines, jatropha, walnut, guava, and even on the skin of animals and humans (21 – 23). Despite its importance, we know very little about the infection mechanism and genetics of *N. dimidiatum*. The best strategy for solving the mystery of a pathogen’s infection mechanism is to obtain information about its genome.

In the study, we aimed to investigate the genetic evolution, gene function, and interactions with the host in *N. dimidiatum* through genome sequencing and comparative analysis. We generated a 43.75 Mb complete genome sequence of *N. dimidiatum*. Through genome annotation and comparative genomic analysis, we showed that *N. dimidiatum* differs from other plant pathogens in terms of disease strategy and disease development. We demonstrated that secondary metabolites and effector proteins play very important roles in the environmental adaptation and pathogenicity of *N. dimidiatum*. These insights contribute to the understanding of the genome and transcriptome of *N. dimidiatum*.

## RESULTS

### The disease cycle of *N. dimidiatum*

**Fig 1:**
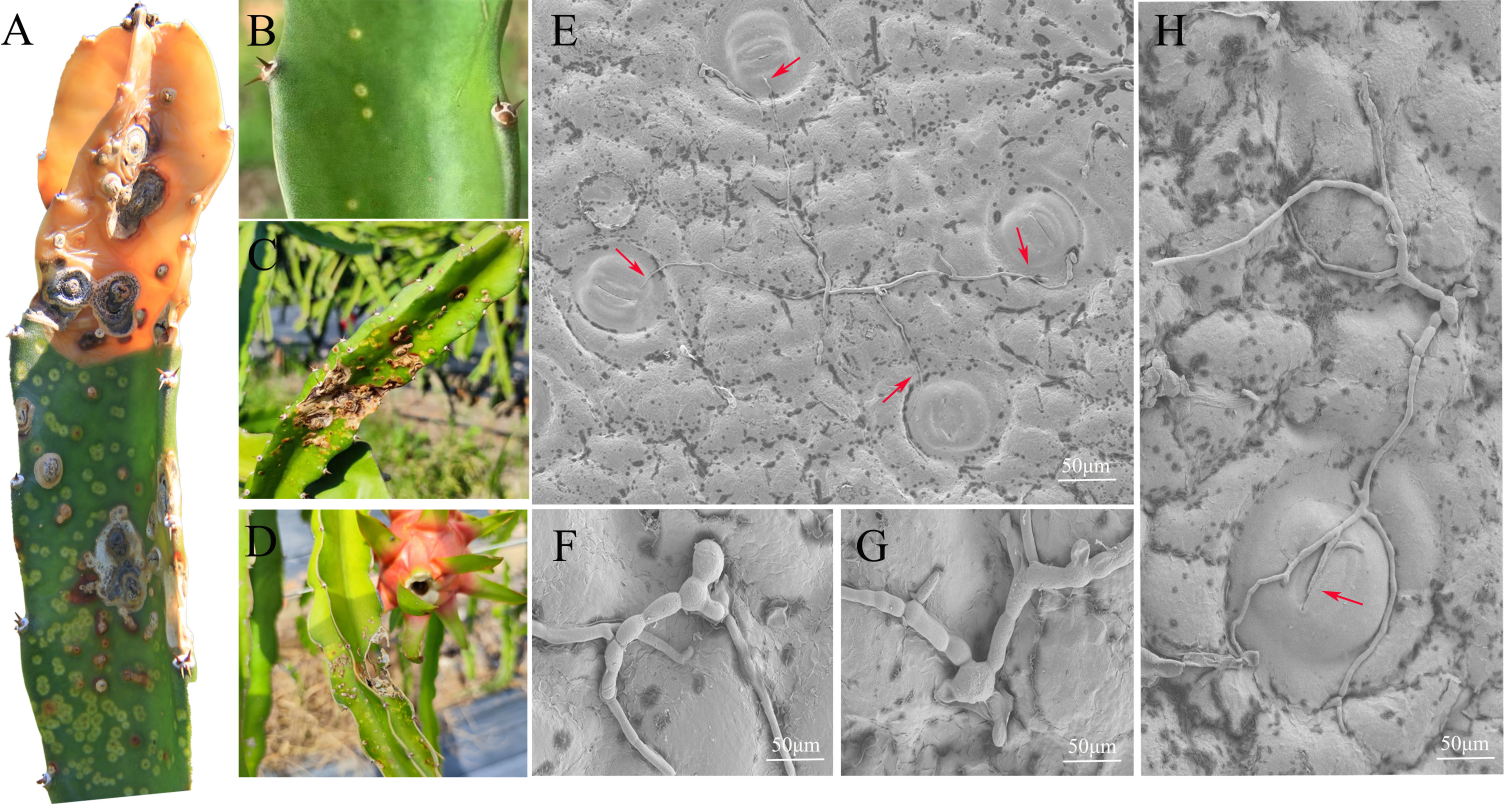
Characteristics of pitaya canker and scanning electron microscopy images of *N. dimidiatum*. (**A**) In the late stage of pitaya stem infection. (**B**) In the early stage of pitaya stem infection, there are small, faded yellow oval-shaped round spots. (**C and D**) In the late stage of pitaya stem infection, the stems wither and dry up when the air is dry. (**E**) Scanning electron microscopy image of *N. dimidiatum* mycelial and spore morphology; mycelium tropism for pitaya stomata. (**F and G**) Attachment structure of *N. dimidiatum* on the surface of pitaya and expanded mycelial attachment points. (**H**) Mycelium entering pitaya stomata.

Microscopic observation revealed that *N. dimidiatum*, along with most of the evaluated fungal pathogens, exhibited similar infection cycles. However, unlike other semi-biotrophic fungi, *N. dimidiatum* did not form an appressorium or infection thread for infecting pitaya, instead infecting through open stomata (Fig. 1; Fig. S1).

### High-quality genome assembly of *N. dimidiatum*

**Fig 2:**
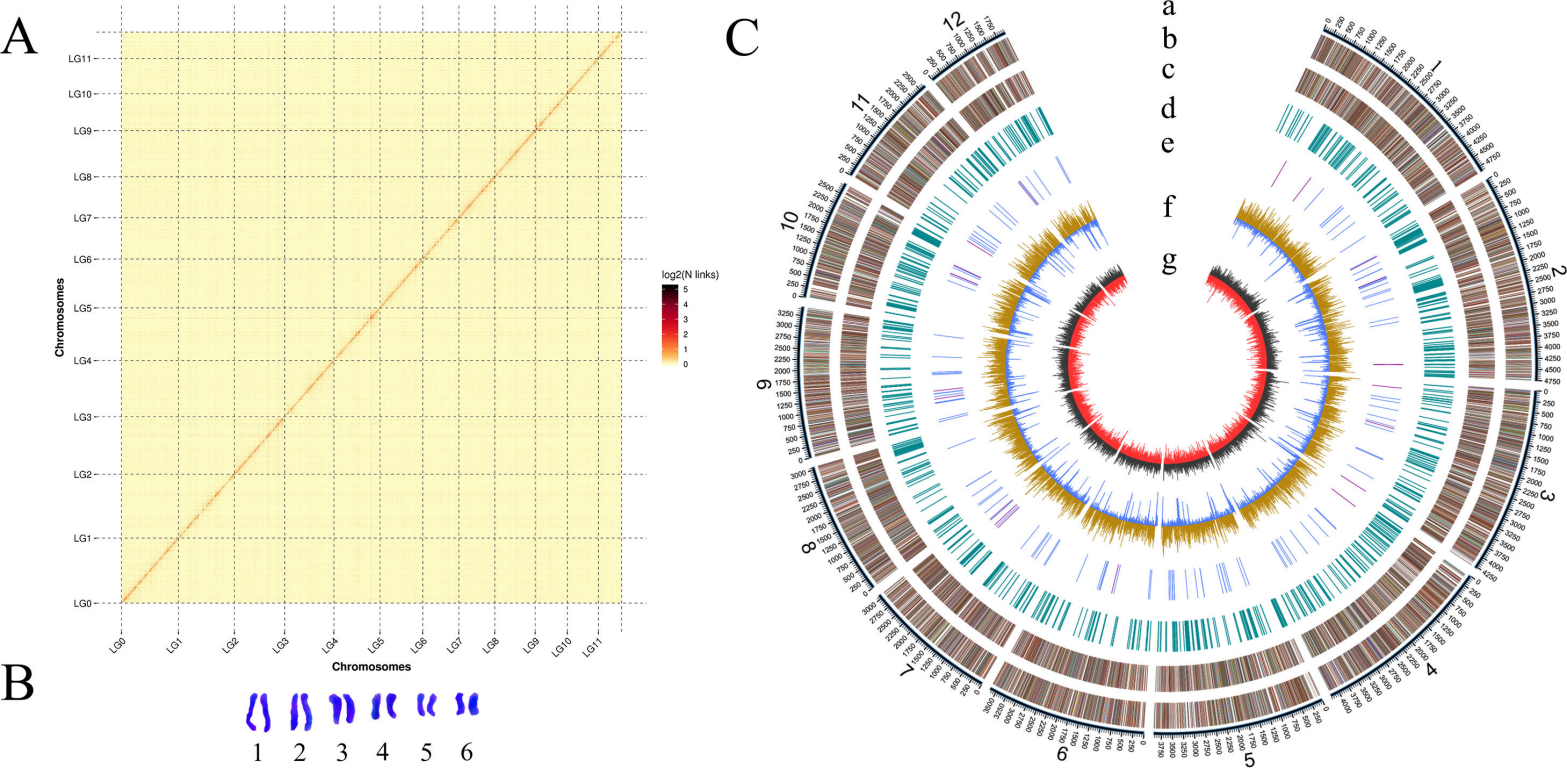
(**A**) Hi-C-assisted assembly of the *dimidiatum* genome. Heatmap showing Hi-C interactions at a resolution of 200 kb and a diagonal pattern of intrachromosomal interactions that may reflect the Rabl configuration of chromatin. (**B**) Cytological analysis of *N. dimidiatum* chromosomes. (**C**) Circos plot of *N. dimidiatum* genome features. (**A**) Twelve largest scaffolds, each scale representing 50 kb; (**B and C**) genes on the forward and reverse genome strands, respectively; different colors represent different COG functional categories; (**D**) repeat sequence; (**E**) tRNAs (blue) and rRNAs (purple); (**F**) GC content. Light yellow indicates a higher GC content than the genome average; the higher the peak, the greater the difference relative to the average GC content. Blue indicates lower GC content than the genome average; (**G**) GC-skew; regions with G > C are shown in dark gray; regions with C > G are shown in red.

A total of 8,869,260,867 of raw data were sequenced by Nanopore and Hi-C platforms (sequencing depth, 186.1×). After the removal of adapters, low-quality reads, and short segments (<2,000 bp), 8,144,071,884 of clean data were obtained. The data were assembled by SMARTdenovo v1.06 into an assembly that consisted of 68 contigs with an N50 of 1.97 Mb (Table S1; Fig. S2). A total of 97.47% of the data could be anchored to the scaffolds of 12 chromosomes, and Fig. 2A shows a heatmap of the assembly. The karyotype analysis examined mitosis in mycelia and meiosis in spores and showed that the chromosomes formed 12 discrete and separate bivalents (Fig. 2B; Fig. S3). A final genome of 43.75 Mb was assembled with a scaffold N50 of 3.93 Mb (Table 1).

**TABLE 1:** Genome features of *N. dimidiatum^a^*

Feature	Value
Assembly features	
Length of scaffolds (bp)	43,759,583
Number of scaffolds	40
N50 scaffold length (bp)	3,925,773
N90 scaffold length (bp)	2,640,097
Maximum of scaffolds (bp)	4,873,947
Scaffold GC content (%)	53.74
Gap length (bp)	2,800
Mapped (%)	96.69
Depth (X)	248.75
BUSCOs (C)	97.59%
Gene models	
Protein-coding genes	12,349
Gene length	26,749,959
Mean number	39,805
Mean exon number	3.22
Mean exon length	601.74
Mean intron length	101.89
Non-protein-coding genes	
rRNAs	21
tRNAs	157
Other ncRNAs	84
Pseudogenes	20

^*a*^ BUSCOs, Benchmarking Universal Single-Copy Orthologs; ncRNAs, non-coding RNAs.

We evaluated the assembly completeness using the next-generation sequencing data return rate and Benchmarking Universal Single-Copy Ortholog (BUSCO) v3.0.2. The former analysis showed that 96.69% of clean reads localized to the reference genome, and the coverage was 99.72%. The latter analysis showed that 97.59% of the BUSCO fungal core genes were identified in the *N. dimidiatum* gene models based on the whole-genome assembly (Table S2). These results indicate a high degree of integrity in the *N. dimidiatum* genome assembly.

Integrated data from ab initio prediction, homologous protein-based prediction, and RNAseq prediction facilitated the prediction of 12,349 high-confidence protein-coding genes in *N. dimidiatum* (Fig. 2C; Fig. S4; Table S3), with an average gene length of 2,166.16 bp, an average coding sequence length of 476.72 bp, and an average exon number of 3.22 (Table 1). There were 157 tRNAs and 20 pseudogenes predicted by RepeatMasker v4.0.6, with a duplicate gene content of 4.56% in the genome (Table S4). Among the protein-coding genes, 96.48% were annotated by functional databases (KEGG, TrEMBL, Swiss-Prot, GO, NR, and COG) (Table S5). In conclusion, we acquired a high-quality genome of *N. dimidiatum*.

### Comparative genomics and evolution

**Fig 3:**
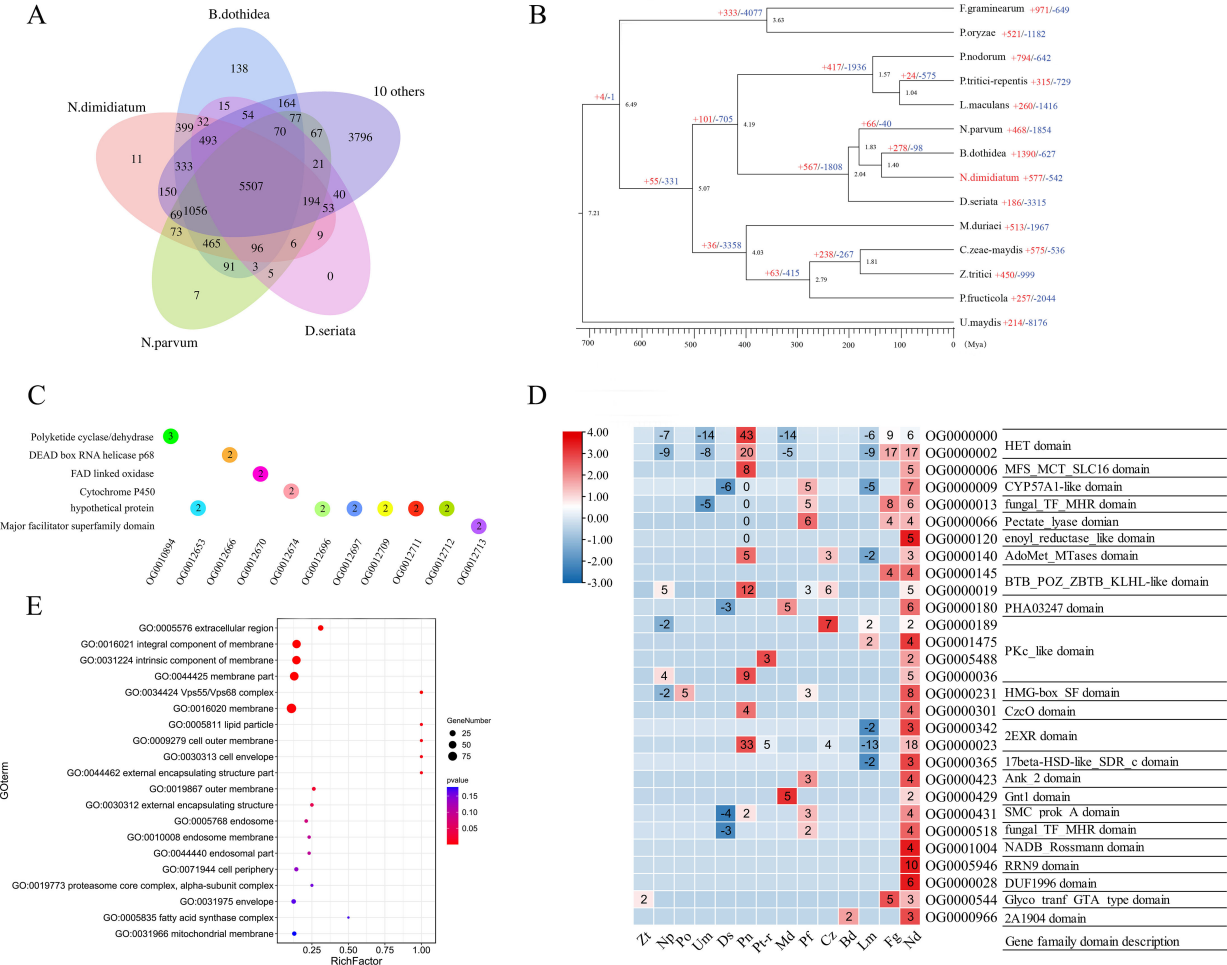
Comparative genomics of *N. dimidiatum* gene families. (**A**) Venn diagram of shared orthologous gene families among 14 pathogenic fungus genomes. (**B**) Inferred phylogenetic tree reconstructed using 1,754 single-copy ortholog families shared by 14 species identified using OrthoFinder. The numbers of expanded and contracted gene families are indicated by plus and minus signs, respectively, before the numbers. (**C**) GO enrichment results of the 11 *N*. *dimidiatum*-specific gene families. (**D**) Functional annotation of the 29 rapidly evolving (expanded) gene families in *N. dimidiatum*. The left panel shows the gene family size among 14 species (x-axis represents species name), and the right panel shows the functional annotation of the gene families. (**E**) GO enrichment results of 577 expanded gene families of *N. dimidiatum*. Source data are provided as a Source Data file.

To better understand the evolutionary relationships between *N. dimidiatum* and other fungi, we used OrthoFinder v2.2.7 to cluster this species with 13 other representative fungi. The analysis revealed that these species shared 1,754 conserved single-copy ortholog families. Phylogenomic analysis reveals that *N. dimidiatum* is evolutionarily close to *Botryosphaeria dothidea* (Fig. 3B). The MCMCtree analysis revealed that *B. dothidea* and *N. dimidiatum* diverged about 22.93 Mya, suggesting a relatively recent speciation event in *Botryosphaeriaceae* (Fig. 3B).

To investigate the evolution of *N. dimidiatum* gene families, we first identified the unique and shared gene families among different fungi. The results reveal that *N. dimidiatum* has 8,946 proteins in shared ortholog families and 487 unique proteins (Fig. 3A). GO and KEGG enrichment analyses of the unique proteins showed that they are involved in regulation of metabolic processes, cellular processes, and single organism processes, as well as catalytic activity and peroxidase activity (Fig. S5; Table S6). We also functionally annotated unique proteins using the Nr database, which showed cytochrome P450 enzymes, glycoside hydrolase, monooxygenase hydrolyze, necrosis-inducing protein, and transcription factor PAP1 (Table S7).

Additionally, *N. dimidiatum* shared 7,466 gene families with *N. parvum* and 8,381 with *B. dothidea*, with only 11 unique gene families found in *N. dimidiatum* (Fig. 3A), with *N. dimidiatum* having only 11 unique gene families. GO enrichment analysis of these unique gene families showed that cellular processes and catalytic synthesis-related pathways were enriched (Fig. 3C).

### Gene family expansions and contractions

*Neoscytalidium dimidiatum* gene family analysis classified 577 and 542 gene families as expanded and contracted, respectively. Moreover, 29 were identified as rapidly evolving families (Fig. 3D). These expanded gene families significantly enriched biological processes, with the most gene members being membrane protein and extracellular protein (Fig. 3E), and these expanded genes occurred for cytochrome P450, followed by oxidoreductases (Fig. S6). Importantly, we found that pectate lyases, lipases, cutinases, necrosis, and ethylene-inducing proteins also exhibited varying degrees of expansion (Fig. S6).

**Fig 4:**
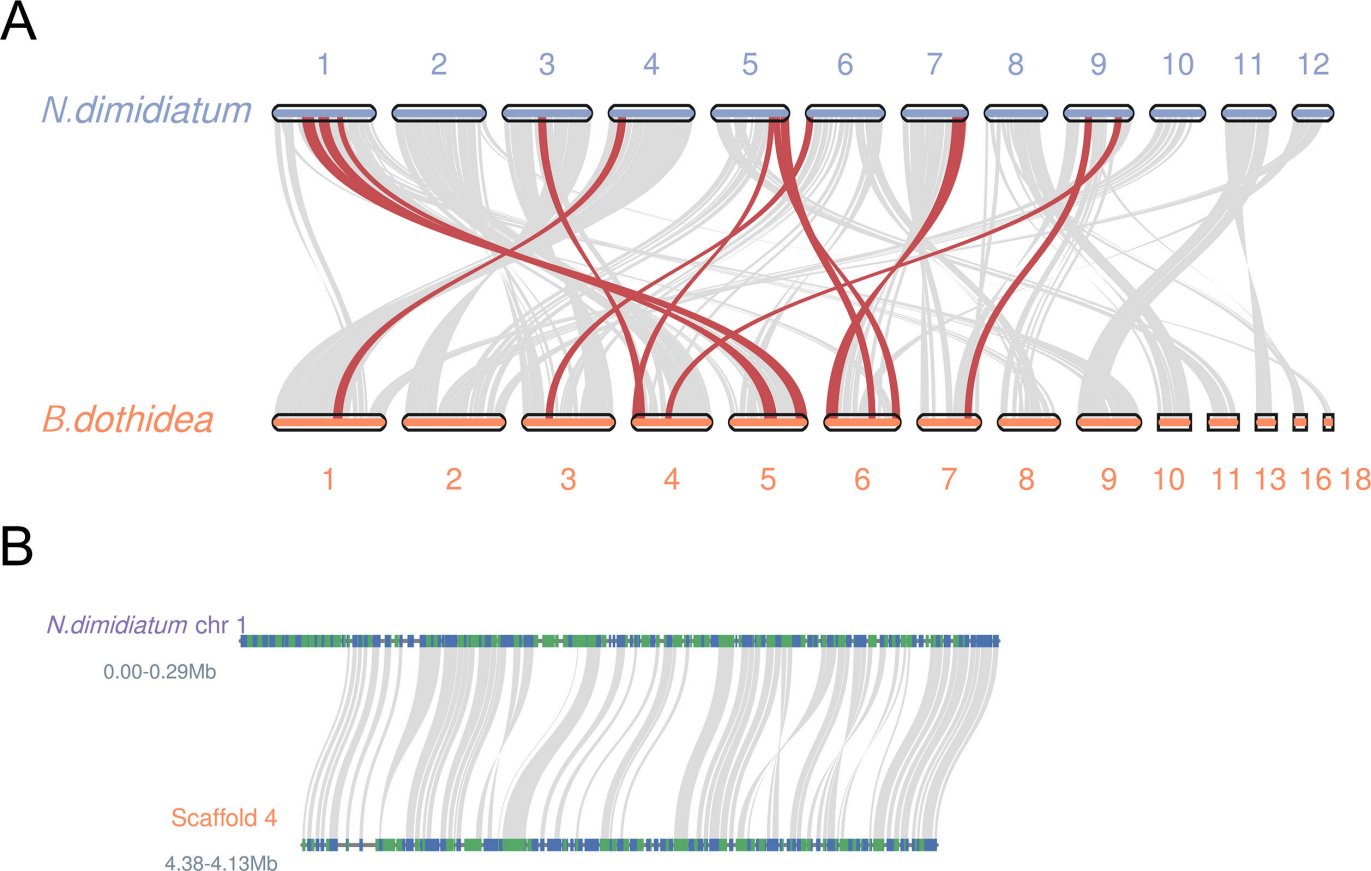
(**A**) Synteny analysis of *N. dimidiatum* and *B. dothidea* using the MCscan subroutine jcvi. The red lines indicate the one-to-one correspondence of the predicted effector proteins of the homologous regions. (**B**) One-to-one relationships between the *N. dimidiatum* and *B. dothidea* genes.

The synteny analysis of the *N. dimidiatum* genome with two *Dothideomycetes* spp. genomes (*B. dothidea* and *N. parvum*) revealed that the *N. dimidiatum* genome exhibits different synteny with different fungi. Of all the above-sequenced genomes, *B. dothidea* exhibited the highest synteny with *N. dimidiatum*. For example, scaffolds 4 and 1 of *B. dothidea* exhibited good synteny with scaffolds 1 and 4 of *N. dimidiatum*, and scaffolds 2 and 3 of *B. dothidea* exhibited good synteny with scaffold 3 of *N. dimidiatum* (Fig. 4A; Fig. S7).

### Gene clusters for secondary metabolite production

The *N. dimidiatum* genome encodes 12 non-ribosomal peptide synthetase (NRPS), 15 PKS (polyketide synthetase), 8 terpene synthetases, 2 β-lactone synthetases, 13 NRPS-like synthetases, 2 NRPS-like/PKS hybrid synthetases, and 1 PKS/terpene hybrid synthetase (Table S8). Secondary metabolite-related core gene annotation revealed that the core gene in 10 of the 53 gene clusters related to the above synthetases was L-aminoadipate-semialdehyde dehydrogenase, which activates α-aminoadipate dehydrogenase in the lysine biosynthesis pathway.

The annotation of the transport proteins of *N. dimidiatum* secondary metabolites also showed that there are many ATP-binding cassette transporters and major facilitator superfamily (MFS) proteins, which are two major classes of transporters in fungi. Their roles in antifungal resistance have been extensively studied in *Candida*, *Aspergillus*, and *Cryptococcus* (24), so this finding reasonably explains the antifungal resistance of *N. dimidiatum*.

Secondary metabolites include pigments that can make the fungi environmentally tolerant and pathogenic (25). They are synthesized mainly through the 1,8-dihydroxy naphthalene (DHN) pathway, with the pigment known as heptaketide naphthopyrene (*YWA1*) being the first intermediate in DHN biosynthesis (25). We found that *N. dimidiatum* has six gene clusters involving PKS genes responsible for pigment production, and the core gene is *YWA1* (Fig. S8A). The results showed that pigments were induced significantly by starvation, suggesting a possible association between pigmentation and resistance to adversity. We further isolated and partially purified these pigments, leading to the identification of two main components: light yellow and dark gray pigments (Fig. S8B).

### Secreted CAZymes

Plant pathogens can disrupt cell walls by secreting carbohydrate-active enzymes (CAZymes), which can help the pathogens to obtain plant nutrients and colonize the plant (8). CAFE v4.2.1 analysis showed that pectate lyases, lipases, and cutinases were strikingly expanded in *N. dimidiatum* (Fig. S6). To investigate the CAZymes secreted by *N. dimidiatum*, we compared the CAZymes of *N. dimidiatum* and 13 other fungi (Fig. S9A and B). *B. dothidea* and *N. dimidiatum* had the highest number of CAZymes, with 1,065 and 968, respectively (Fig. S9A). *N. dimidiatum* also had the highest number of PCWDEs, with 18 (18.9%) pectin lyases and 31 (32%) lipases, which far exceeded the average (Fig. S9B). These results demonstrate that *N. dimidiatum* has a highly diverse repertoire of CAZymes, which may contribute to its ability to cause canker disease in pitaya plants.

**Fig 5:**
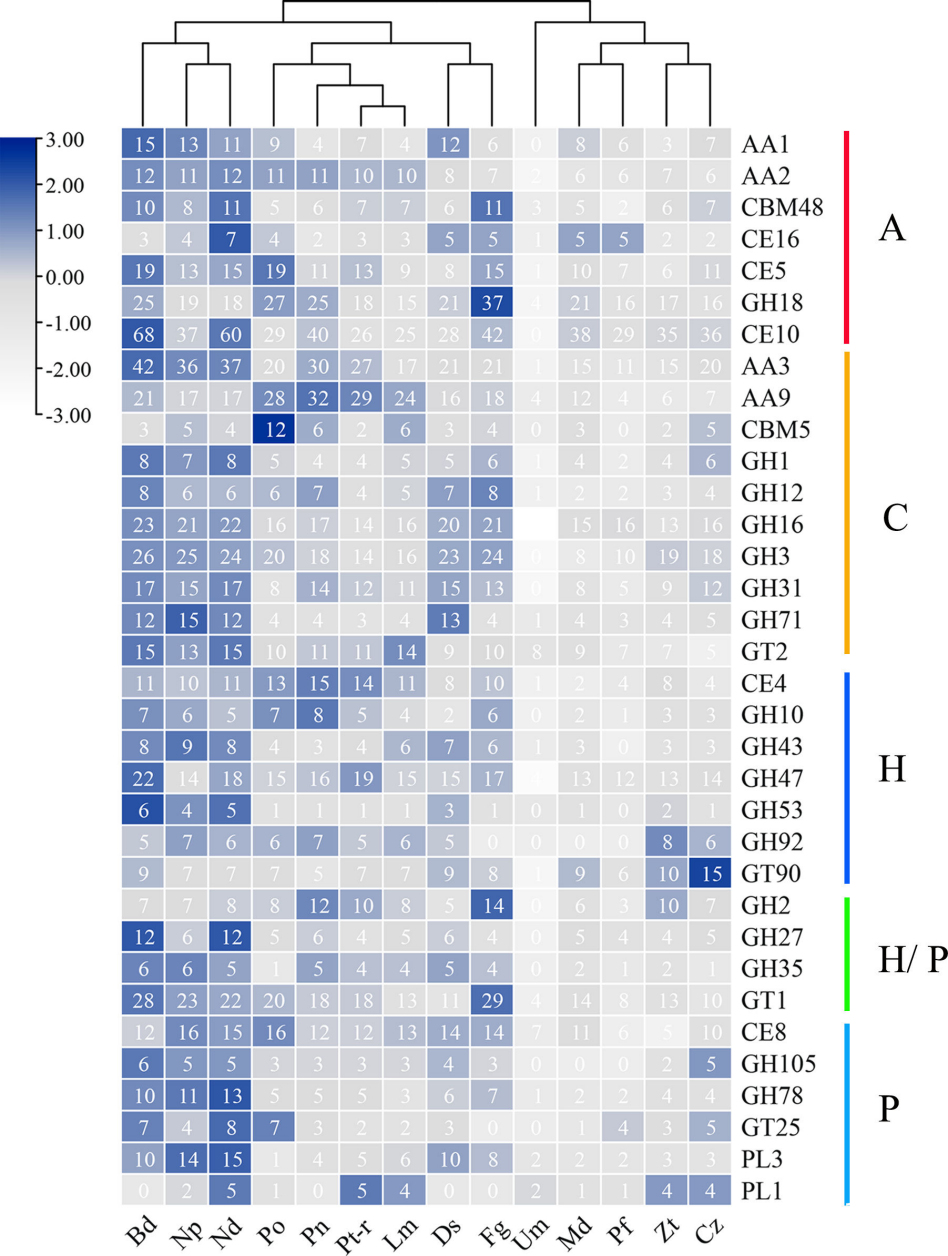
Comparison of CAZymes between *N. dimidiatum* and 13 other fungal species. The numbers of members for each genome are shown. Bd = *Botryosphaeria dothidea*; Np = *Neofusicoccum parvum*; Nd = *Neoscytalidium dimidiatum*; Po = *Pyricularia oryzae*; Pn = *Parastagonospora nodorum*; Pt-r = *Pyrenophora tritici-repentis*; Lm = *Leptosphaeria maculans*; Ds = *Diplodia seriata*; Fg = *Fusarium graminearum*; Um = *Ustilago maydis*; Md = *Myriangium duriaei*; Pf = *Peltaster fructicola*; Zt = *Zymoseptoria tritici*; Cz = *Cercospora zeae-maydis*.

Additionally, *B. dothidea*, *N. dimidiatum*, and *N. parvum* were highly enriched in cellulase, hemicellulase, and pectin lyase genes (Fig. 5). Pectin lyase was expanded the most in *N. dimidiatum* (PL1, PL3, GH78, and GT25) (Fig. 5), indicating that *N. dimidiatum* has a strong ability to degrade pectin (26). *Botryosphaeria dothidea*, *N. dimidiatum*, and *N. parvum* had similar numbers of expanded CAZymes, indicating that they have similar lifestyles in some respects, but there were differences in the numbers of cellulases, hemicellulases, and pectin lyases, which might explain the differences in pathogenicity (Fig. 5). Of the annotated CAZymes, the CE10 family of carbohydrate esterases expanded the most in *B. dothidea* and *N. dimidiatum*. Additionally, the GH27 family of glycoside hydrolases has evolved a carbohydrate-binding module 1 structural domain, which helps them to bind to cellulose (Fig. 5).

### Secretome and potential effectors

During infection of a host by a pathogenic fungus, the pathogen secretes a series of proteins to promote colonization and infestation (27). Therefore, the identification and functional annotation of pathogen-secreted proteins are of particular interest for the identification of effectors. In *N. dimidiatum*, 121 effectors were predicted, with 68–354 amino acids and molecular weights of 7.077–36.383 kDa (Fig. S10). Of these, 63% were not annotated using the Nr database; however, pectin lyases accounted for 7%, necrosis and ethylene-inducing proteins for 3%, cutinases for 1%, and cysteine-rich secreted proteins for 1% (Fig. S11). These candidate effectors may play important roles in infection and colonization, promoting pathogenicity in plants.

The 121 effectors were classified into 10 clusters by the phylogenetic tree and gene annotation (Fig. S12) and evenly mapped onto the chromosomes (Fig. S13). Interestingly, genes with similar functions were grouped into the same cluster but distributed across different chromosomes. For example, pectin lyases were assigned to cluster II but located on chromosomes 4, 5, 6, 7, and 9; necrosis and ethylene-inducing proteins were assigned to cluster X but distributed on chromosomes 1, 3, 5, 6, and 8 (Fig. S13). The predicted effectors are involved in different aspects of plant infestation and colonization and may have complementary functions.

### Functional analysis of effectors

**Fig 6:**
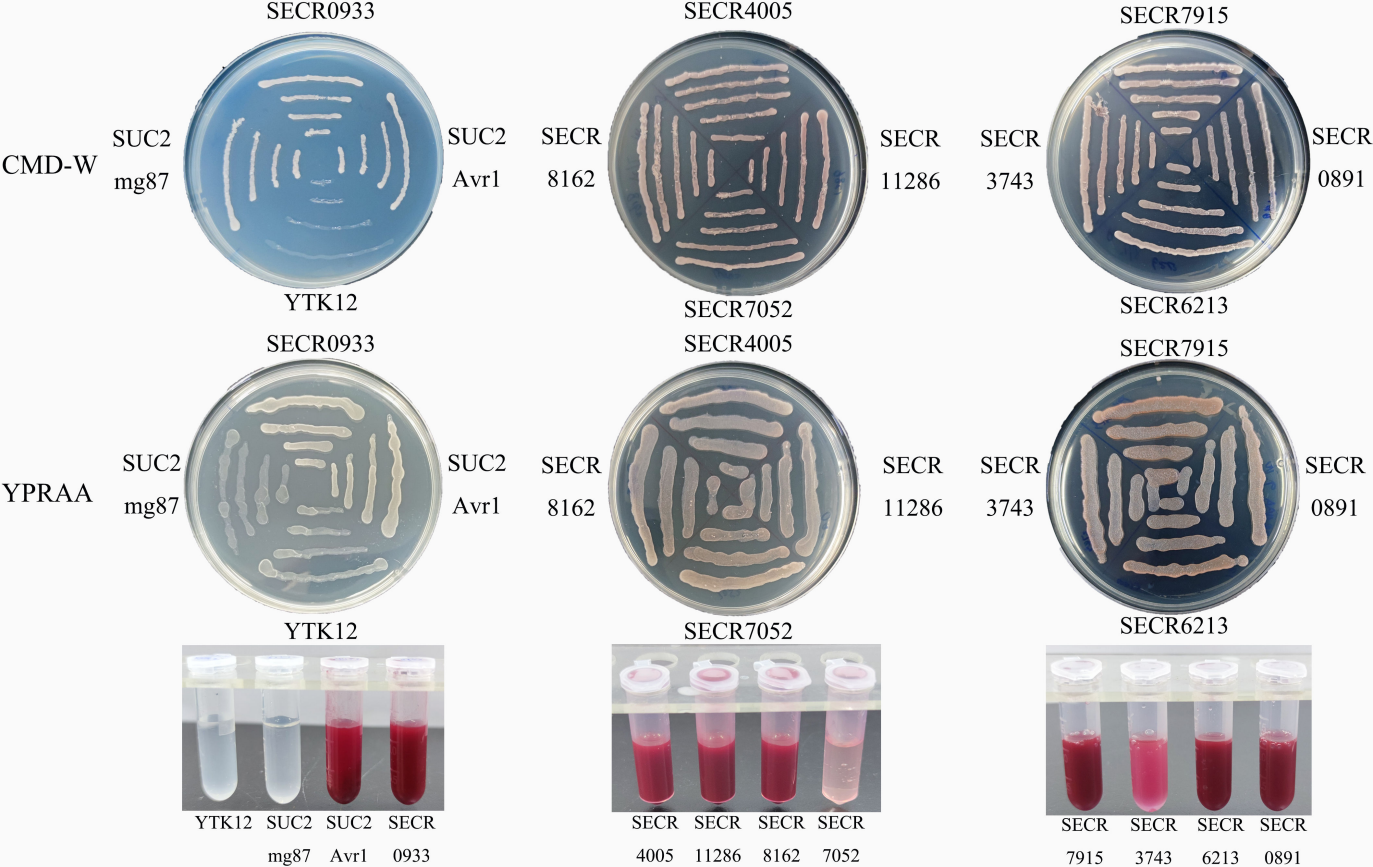
The predicted effector proteins are secreted proteins. Signal peptide-coding sequences of nine effector proteins were each fused into a pSUC2 vector and then transformed into the YTK12 yeast strain. Avr1b signal peptide and the first 25 amino acids of Mg87 were used as positive and negative controls, respectively. The growth of the yeast strain on CMD-W (minus Trp) medium (first row) confirmed that the vector was transformed into the yeast strain. Growth on YPRAAA medium (second row) and triphenyl tetrazolium chloride color change (third row) confirmed the secretion brought about by the effector protein signal peptide.

Of the 293 predicted effectors, nine were randomly selected and found to have secretory function based on a yeast secretion assay. These effectors were transformed into the YTK12 yeast strain to express their signal peptides, and they exhibited secreted invertase activity like the positive control (Fig. 6). These results indicate that the predicted signal peptide (SPs) of the nine putative *N. dimidiatum* effectors are functional to direct these proteins to the secretory pathway and that these proteins are bona fide secreted proteins.

**Fig 7:**
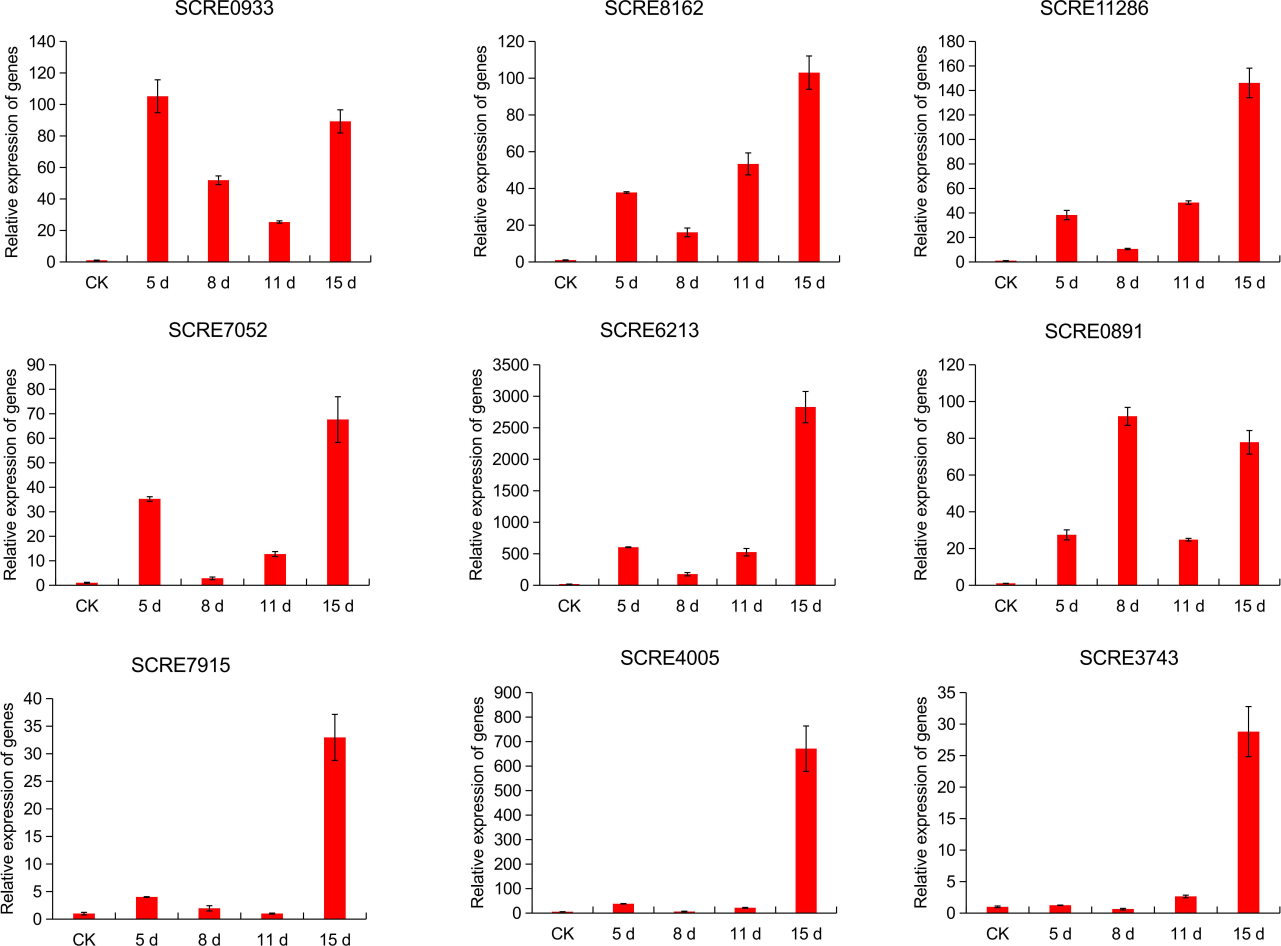
Expression profiles of nine predicted effector protein-coding genes during *N. dimidiatum* infestation of pitaya. Infected pitaya branches were collected at 0, 5, 8, 11, and 15 dpi for gene expression analysis by real-time quantitative reverse transcription-polymerase chain reaction. Data are mean ± SD. The gene expression patterns represent three independent repeats with similar results.

To reveal the effector protein-coding genes regulated during *N. dimidiatum* infection, real-time quantitative PCR (RT-qPCR) was used to assess the expression of the nine secreted effector protein-coding genes at different time points during *N. dimidiatum* infection. The genes were divided into two groups based on their expression patterns: four genes (*SCRE0933*, *SCRE8162*, *SCRE11286*, *SCRE7052*, *SCRE6213*, and *SCRE0891*) showed early up-regulation during infection, while the remaining five genes (*SCRE7915*, *SCRE4005*, *SCRE3743*, *SCRE0933*, and *SCRE8162*) showed up-regulation at 15 dpi (Fig. 7). These results suggest that the nine secreted protein genes are differentially regulated during fungal infection.

**Fig 8:**
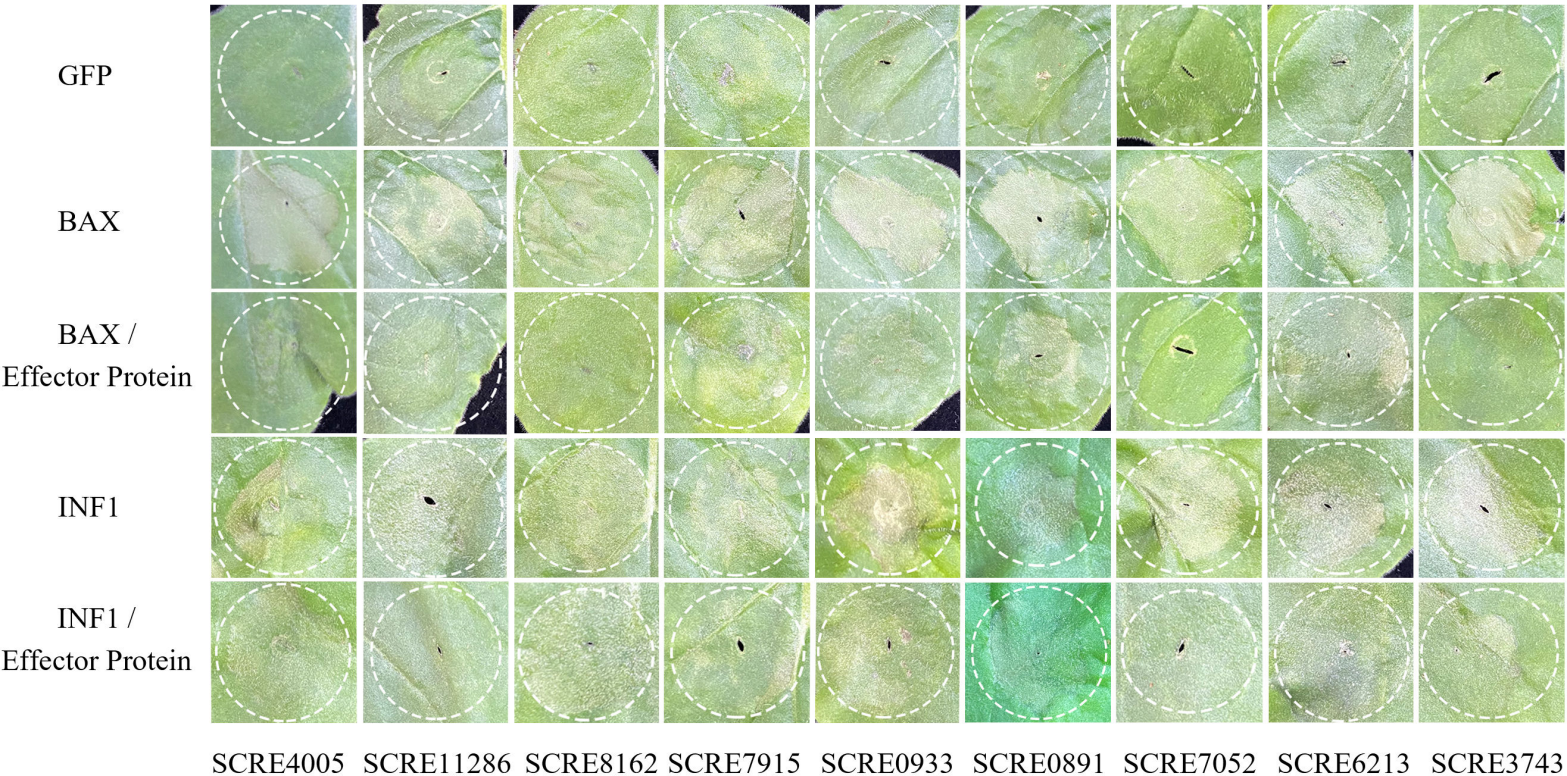
Putative effectors in *N. dimidiatum* inhibit cell death symptoms in *Nicotiana benthamiana*. Green fluorescent protein was used as the negative control. *Agrobacterium* cells carrying a BAX (OD600 ∼ 0.3) or INF1 (OD600 ∼ 0.3) construct and/or an effector construct (OD600 ∼ 0.3) were equally mixed and infiltrated into 4-week-old *N. benthamiana* leaves with needleless syringes. Cell death symptoms were recorded at 3–4 dpi.

Furthermore, we investigated the function of the effectors. We constructed nine effector constructs and transiently expressed them in *Agrobacterium* GV3101, and we found that the nine effectors strongly inhibited cell death triggered by BAX or INF1 (Fig. 8), implying that they may contribute to early infection in pitaya by inhibiting defense-related cell death.

## DISCUSSION

We found that *N. dimidiatum* has the same infection cycle as most fungi but did not form an appressorium or a penetration peg. More importantly, *N. dimidiatum* entered the host plant’s intercellular space via stomata.

*N. dimidiatum* is an opportunistic pathogenic fungus that has become highly threatening to pitaya, and it seems to have tools in its genome to cope with environmental climate changes and a wide variety of plants. In this study, we generated a complete *N. dimidiatum* genome sequence using long reads generated by Nanopore and Hi-C sequencing. We assembled the genome of *N. dimidiatum* into 12 chromosomes. The genome size and chromosome assembly showed that the *N. dimidiatum* genome is diploid. This represents important information for comparative genomic studies of the family *Botryosphaeriaceae*. We found that *N. dimidiatum* and *B. dothidea* likely diverged approximately 22.93 Mya. More importantly, the results advance our understanding of the evolution of *N. dimidiatum*.

The genome and gene evolution of fungi enable them to exist in diverse environments (28). In this study, members of *N. dimidiatum* showed significant expansion and contraction, and among these genes’ cytochrome P450 exhibited the most significant expansion (Fig. S6). Consistent with the genomes of *B. dothidea*, these numbers are higher than for the other fungi with which we compared them (29). P450 can facilitate fungal adaptation to the environment by altering potential chemicals in the environment, and more importantly, it is closely related to fungal physiology (10). Furthermore, the MFS plays an important function in fungal spore formation, intercellular communication, and defense against plants (24). Both *B. dothidea* and *N. dimidiatum* have larger MFS families than other fungi (Fig. S6) (30).

Among the compared species, there is a significant divergence in the genomic composition of CAZymes, particularly between evolutionarily distant species (Fig. S10). Such species as *N. dimidiatum*, *B. dothidea*, and *N. parvum* exhibit a greater abundance of CAZyme genes compared to other tested species, enabling them to effectively disrupt plant cell walls and successfully infect host plants. Additionally, among the six categories of CAZymes, lipases, cutinases, and pectate lyases were abundant in both *N. dimidiatum* and *B. dothidea* and indicated the expansion of specific gene classes. These results suggest that *N. dimidiatum* and *B. dothidea* have evolved increased virulence and resistance to plant defense compounds, which could be related to their specialized mechanisms for evading host defenses and promoting their own growth and survival.

In the comparative genomics analysis, it was found that AA9 lytic polysaccharide monooxygenase which is known for its ability to degrade plant cellulose (31), was significantly enriched in *N. dimidiatum*, *P. oryzae*, and *P. tritici-repentis* (Fig. 5). Additionally, the AA1 gene family, which encodes lignin-degrading enzymes, and the CE5 gene family, which encodes cutinases, showed varying degrees of expansion in these three strains (11). Moreover, the glycoside hydrolase (GH) gene family, which may contribute to the increased virulence of *Botryosphaeriaceae* and their broad host range, was also abundant in all three tested species (11, 16, 17). It is noteworthy that *N. dimidiatum* and *B. dothidea* exhibited expansion in the PL3 (pectate lyase) gene family (Fig. 5). This gene family encodes alpha-D-galacturonan lyase, which cleaves alpha-D-galacturonan (27, 32). These findings suggest that the enhanced virulence of *N. dimidiatum* may be due in part to its ability to enzymolysis pectin, a key component of pitaya cell walls. This information could provide valuable insights into the mechanisms underlying the success of this fungal pathogen in its interactions with its host and potential targets for developing new control strategies.

In fungi, melanin production is primarily mediated by the DHN and L-3,4-dihydroxyphenylalanine pathways (25), which serve to provide protection against various adversities and act as virulence factors for the fungus. We found that the *N. dimidiatum* genome encodes a variety of PKSs responsible for melanin production, and we also found that *N. dimidiatum* can produce melanin. Previous research has suggested that melanin produced by *Cercospora sojina* is associated with virulence (18), while in *Magnaporthe oryzae* and *Colletotrichum* spp., melanization is a critical step in the accumulation of high turgor pressure within the infection structure appressorium to penetrate the host epidermis (25). Moreover, we isolated two melanins, light yellow and gray black, through starvation treatment of *N. dimidiatum*. These results suggest that melanization may play an important role in the pathogen’s ability to resist adversity.

Some of the effectors produced by phytopathogens are recognized by plant immune receptors, thus triggering the plant immune response (19). We found that the closer the evolutionary relationship, the closer the number of effector proteins; e.g., the number of effector proteins of *N. dimidiatum*, *N. parvum*, and *B. dothidea* were 121, 124, and 152, respectively. The annotation results of *N. dimidiatum* effector proteins showed that these proteins include pectate lyases, cutinases, necrosis and ethylene-inducing proteins, and cysteine-rich secretory proteins, which are known to be virulence factors in other fungi (10, 26, 32, 33). These findings suggest that the pathogenicity of these fungi may be related to their ability to interact with host plants through the production of specific effector proteins. We also verified that these effectors are secreted and suppress plant immune responses during fungal invasion by using yeast secretion assays and cell death inhibition assays in *N. benthamiana*. These data demonstrate that *N. dimidiatum* can probably deploy effectors to promote infection.

In summary, we report a complete *N. dimidiatum* genome sequence (99.72% of the estimated genome) obtained using Nanopore and Hi-C sequencing. Based on genome assembly, annotation, and comparison to other fungal genomes, we hypothesize that specific CAZymes (PCWDEs), secondary metabolites, and other effectors of *N. dimidiatum* help it to adapt to pitaya and other hosts. The results provide a foundation for future research on *N. dimidiatum* and the control of pitaya canker.

## MATERIALS AND METHODS

### Strain and DNA extraction

*N. dimidiatum* was originally isolated at a pitaya plantation in Ledong (N34°24′, E108°39′), Hainan Province, in 2015. It is very aggressive on pitaya stems and is stored at the Key Laboratory for the Sustainable Utilization of Tropical Bioresources, Hainan, China. Isolated hyphae were grown on potato dextrose agar (PDA) medium at 28°C. Thereafter, the mycelia were removed from the media and ground in liquid nitrogen. Genomic DNA was extracted using a modified cetyltrimethylammonium bromide method.

### Oxford Nanopore sequencing library construction and sequencing

To generate long reads by Nanopore sequencing, a fully automated BluePippin (Sage Science Inc., Beverly, MA, USA) was first used to select DNA fragments of ∼15 µg (30–80 kb), and a library was constructed using an SQK-LSK109 ligation-based sequencing kit (NEB, USA). Next, the library was sequenced using three R9.4 flow cells for 48 h with a PromethION DNA sequencer (Oxford Nanopore, Oxford, UK). The offline sequencing data were in the binary FAST5 format, containing all the original sequencing signals. These data were converted to the fastq format, and then adapters, low-quality reads, and short segments (<2,000 bp) were removed to obtain clean reads.

### Hi-C library construction and sequencing

*N. dimidiatum* Hi-C libraries were generated by BioMarker Technologies Company, as described previously (34). The libraries underwent quality control (HiSeq Control Software v2.2.58) and were sequenced on an Illumina HiSeq X Ten platform.

### *De novo* genome assembly

Canu v1.7 (30) was used to correct the raw Nanopore reads, and wtdbg2 v2.5 (35) was used to assemble error-corrected reads to obtain the contigs. The next-generation sequencing reads were aligned to the error-corrected contigs by BWA-MEM, and error correction was performed by Pilon to obtain the final version of the contigs (36). Next, the Hi-C paired-end clean reads were aligned to the final version of the contigs using Juicer v1.5 (37) to obtain an interaction matrix. The position of the contigs was visually corrected based on Hi-C heatmaps using Juicebox v1.11.08. Finally, the completeness of the genome assembly was evaluated by using BUSCO v3.0.2 to conduct a search against the fungi_odb9 data set of 290 conserved core genes (38).

### Annotation of repetitive sequences

LTR_FINDER RepeatModeler v1.0.5 (39) was used to identify the repeats. The database was classified using the PASTEClassifier and then merged with the Repbase database to create the final repetitive sequence database (40). The repetitive sequences were then predicted based on the final repetitive sequence database using RepeatMaker v4.0.6 (41).

### RNAseq data analysis

We collected *N. dimidiatum* hyphae cultured in PDA medium for 5 days and extracted RNA using the Trizol method, with three biological replicates per sample. A library was prepared and sequenced on the Illumina HiSeq X Ten platform. After generating RNAseq reads, they underwent adapter removal, and low-quality bases were trimmed using fastQC v0.12.6 (https://www.bioinformatics.babraham.ac.uk/projects/fastqc/). Thereafter, 682,606 bp of clean data remained for gene prediction and expression analysis. The clean reads were mapped to the *N. dimidiatum* genome using HISAT2 v2.0.5 (42) with default parameters. Following the alignment, the Summarized Experiment object was generated using StringTie v1.3.3b (43).

### Protein-coding gene prediction and function annotation

To annotate the protein-coding genes of *N. dimidiatum*, we used a combination of ab initio gene prediction, homologous protein-based gene prediction, and RNAseq-based gene prediction. EvidenceModeler v1.1.1 was used to integrate the three sets of results to obtain a comprehensive, non-redundant collection of genes (44, 45). Additionally, COG, KEGG, and GO analyses were conducted.

### Genomic comparisons and visualization

We aligned the protein sequences of *N. dimidiatum* with those of 13 other fungal species using OrthoFinder v2.2.7 (46). CAFE v4.2.1 (47), with default parameters, was used to identify the expanded and contracted gene families for each species. Using the MCscan subroutine jcvi v1.0.1, the gff file was converted to a bed file, duplicates were removed, the DNA and protein sequences were extracted, and Python was used for synteny analysis.

### Phylogenetic analysis and divergence time estimation

To determine the phylogenetic relationships between *N. dimidiatum* and 13 other fungal species, after downloading their protein sequences and aligning them using OrthoFinder v2.2.7, we used MUSCLE v3.8.31 (48) to calibrate them. A phylogenetic tree was then constructed by the maximum likelihood method using RAxML v8.2.10 under the GTRGAMMA model (49). The divergence time among the species was calculated using the MCMCtree program in PAML v4.8 and then calibrated with the divergence time from TimeTree (50).

### Predicting effectors in the secretome

Protein-coding sequences from *N. dimidiatum* were scanned for possible signal peptides using SignalP v5.0 (51). The amino acid sequences containing predicted signal peptides were scanned for transmembrane proteins using the TMHMM program (52). Effectors in the secretome were identified using the machine learning program EffectorP v3.0 (53).

### Comparative analysis of CAZyme and secondary metabolism-related genes

To annotate CAZymes, HMMER v3.0 (54) packages were used to conduct a homology search. Family-specific hidden Markov model profiles were downloaded from the dbCAN database. The executable file hmmscan and the hmmscan-parser script provided by dbCAN were used to generate and extract the search results, respectively.

To annotate genes involved in the biosynthesis of secondary metabolites, the modules of individual NRPS and polyketide synthetase (PKS) proteins were identified by searching the antiSMASH database. The secondary metabolite-related core genes were then annotated using standalone BLAST against the Swiss-Prot database.

### Karyotype analysis

A sterile coverslip was inserted into a culture dish containing PDA medium, and *N. dimidiatum* mycelia were used to inoculate the medium at 28°C until the mycelia grew on the coverslip. The coverslip was removed with tweezers, fixed with Carnoy’s Fluid for 24 h at room temperature, rapidly dried using an alcohol flame, placed in 75% and then 95% alcohol, placed in lyticase for 1 h at 37°C, and treated with a modified carbon-fuchsin solution (Solarbio, Beijing) for 10 min. The excess solution was absorbed with filter paper. Observation and calculations were carried out using microscopy (IX73, Olympus, Japan).

### Yeast secretion assay

A yeast secretion assay was used for secretion verification (19).

### Real-time quantitative PCR

Gene expression was determined by RT-qPCR using an ABI 7500 sequence detection system (Applied Biosystems, Foster City, CA, USA). The reaction mixture (20 µL) contained 0.5 µL cDNA, 10 µL SYBR Premix Ex Taq (Vazyme), and 0.2 µM forward and reverse primers. The PCR conditions were as follows: initial denaturation at 95°C, followed by 40 cycles of denaturation at 95°C for 5 s, and annealing at 60°C for 30 s. A melting curve analysis was carried out at 60–95°C, and the temperature increment was 1°C to verify the specific amplification. *Tubulin* was used as an internal control (55). The primer sets used for qRT-PCR and RT-PCR are listed in Table S9.

### *Agrobacterium*-mediated transient gene expression

BAX/INF1 (which induce cell death) gene constructs and the effector gene constructs were produced using PGR107 vectors and transformed into *Agrobacterium* GV3101. For transient expression in *N. benthamiana*, overnight-cultured *Agrobacterium* strains were collected, washed twice with distilled ddH_2_O, and resuspended in 10 mM MgCl_2_ with 150 µM acetosyringone and 10 mM MES (pH 5.7) to suitable concentrations at room temperature for 3 h. *Agrobacterium* cells carrying a BAX (OD_600_ ~ 0.3) or INF1 (OD_600_ ~ 0.3) construct and/or an effector construct (OD_600_ ~ 0.3) were then mixed evenly and infiltrated into the leaves of 4-week-old *N. benthamiana* with a needleless syringe. Cell death symptoms were recorded at 3–4 days post-infiltration (dpi).

## Data Availability

The *Neoscytalidium dimidiatum* genomes sequenced in this study were deposited in the GenBank database with accession number PRJNA1018469.

## SUPPLEMENTAL MATERIAL

Supplemental figures spectrum.02733-23-s0001.pdf Fig. S1 to S13.

Table S1 spectrum.02733-23-s0002.xlsx Genome assembly result statistics.

Table S2 spectrum.02733-23-s0003.xlsx Next-generation sequencing data return rate.

Table S3 spectrum.02733-23-s0004.xlsx Statistics of gene annotation in the *N. dimidiatum* genome.

Table S4 spectrum.02733-23-s0005.xlsx Statistics of the repeats in the *N. dimidiatum* genome.

Table S5 spectrum.02733-23-s0006.xlsx Statistics for functional annotation.

Table S6 spectrum.02733-23-s0007.xlsx Functional enrichment of the 487 unique genes in *N. dimidiatum*.

Table S7 spectrum.02733-23-s0008.xlsx Nr database for functional annotation of 209 proteins of *N. dimidiatum*.

Table S8 spectrum.02733-23-s0009.xlsx Annotation of putative back-bone genes of secondary metabolisms.

Table S9 spectrum.02733-23-s0010.xlsx Primer sequences used for this study.

ASM does not own the copyrights to Supplemental Material that may be linked to, or accessed through, an article. The authors have granted ASM a non-exclusive, world-wide license to publish the Supplemental Material files. Please contact the corresponding author directly for reuse.
