# Supplementary material for: Whole-genome sequencing and comparative genomics reveal the potential pathogenic mechanism of Neoscytalidium dimidiatum on pitaya
Source: Microbiol Spectr. 2023 Nov 21;11(6):e02733-23. doi: 10.1128/spectrum.02733-23 (PMC10714984; doi:10.1128/spectrum.02733-23)
Supplement: Supplemental figures — Fig. S1 to S13. [file spectrum.02733-23-s0001.pdf]

## Supplemental Figures and their legends

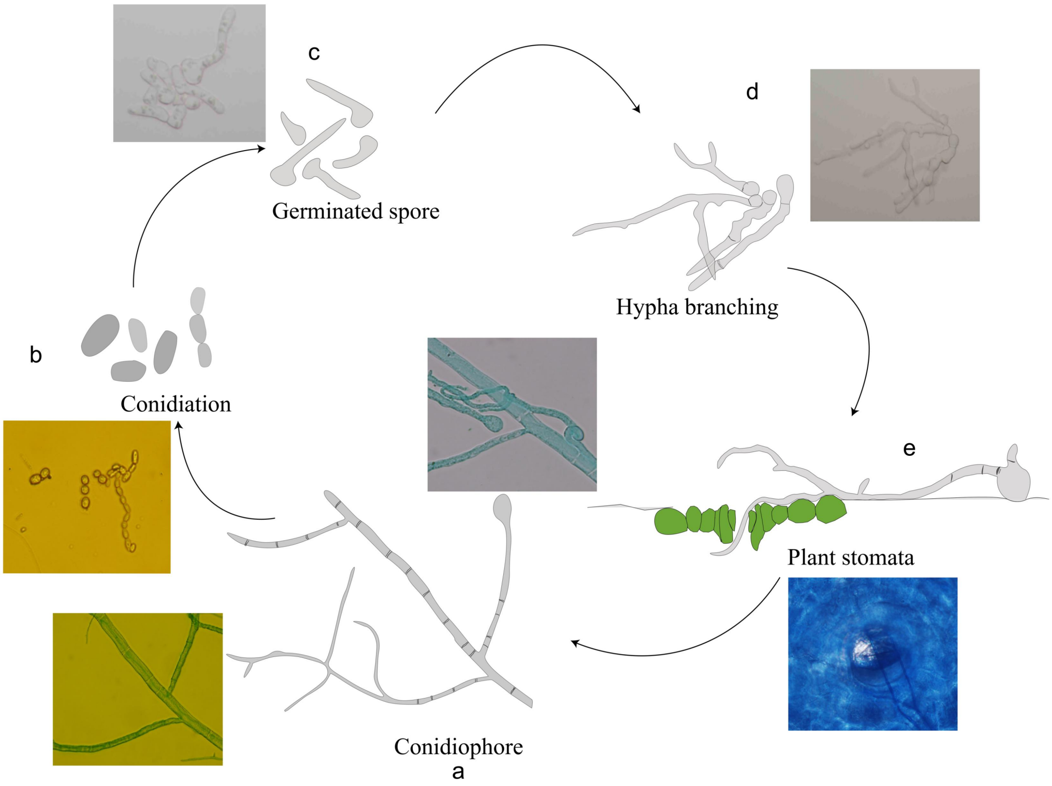


**FIG S1** Life cycle and morphology of *N. dimidiatum* in different periods. (a) The mycelium turns from white to gray black to black again in extreme, lack of food or lack of space environment, and the mycelium produces conidia, meristem arthrospores and even chlamydospore. (b) Meristem arthrospores and chlamydospore. (c) One end of the spore begins to expand to secrete a sticky substance on the pitaya for colonization, and the other end elongates to form a germtube. (d) This spore will branch as it continues to grow, the germtube branches to form young mycelium. (e) Young mycelium can infest pitaya through stomata.

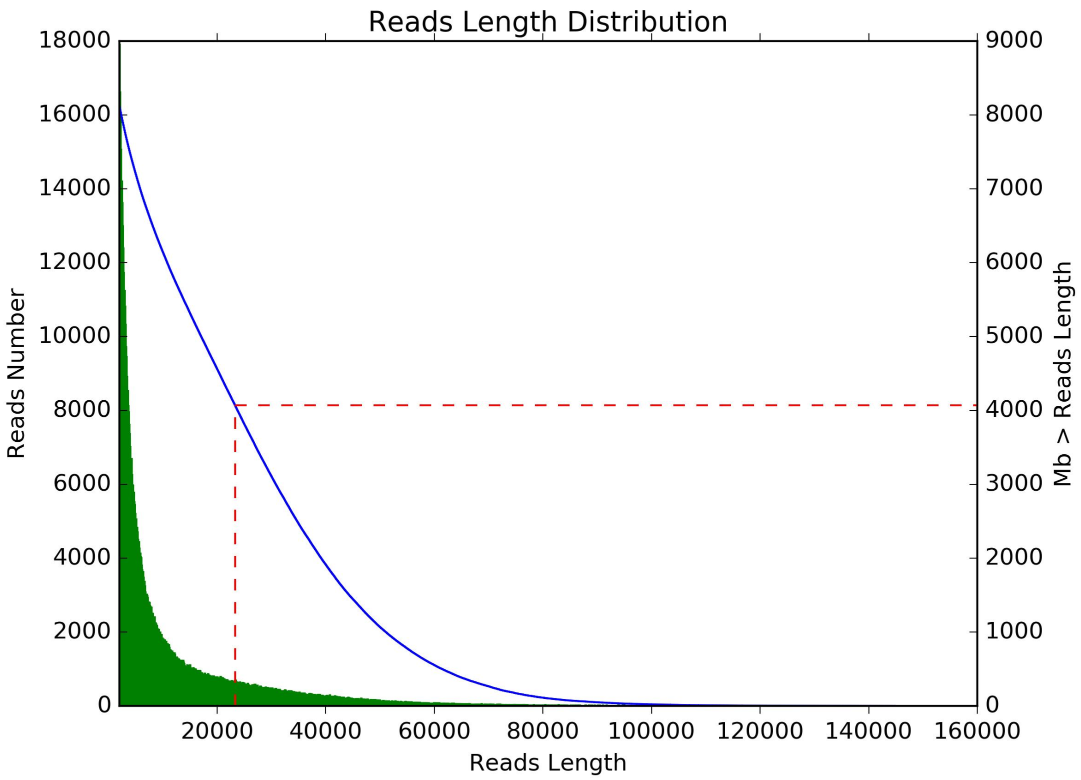


**FIG S2** Distribution of data length. The horizontal coordinate represents the length of the reads (bp); the left vertical coordinate represents the number of reads, corresponding to the green bar graph; the right vertical coordinate represents the total number of bases (Mb) contained in reads larger than the corresponding length, corresponding to the blue curve; the red dashed line indicates the N50 length of the reads.

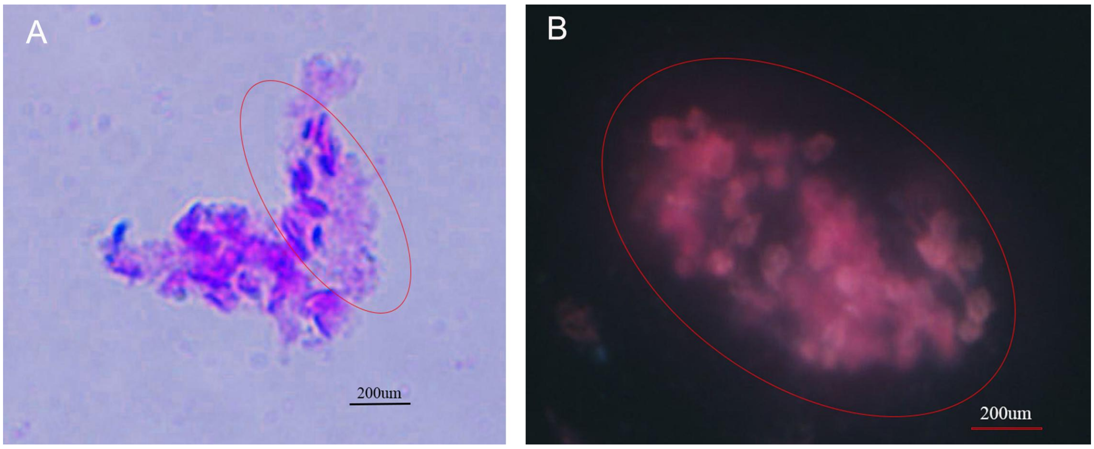


**FIG S3** Cytological analysis of *N. dimidiatum*. (A) Pachytene, chromosomes become shorter and thicker. The karyotype analysis revealed that there are 24 chromosomes in the cell. (B) Anaphase I, homologous chromosomes move to the opposite poles under the traction of the spindle.

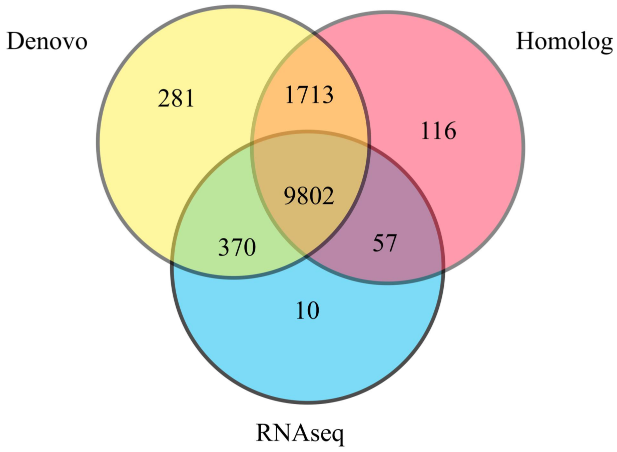


**FIG S4** Statistical plots of prediction results of Ab initio based, RNA-seq based and Homology based.

A

B

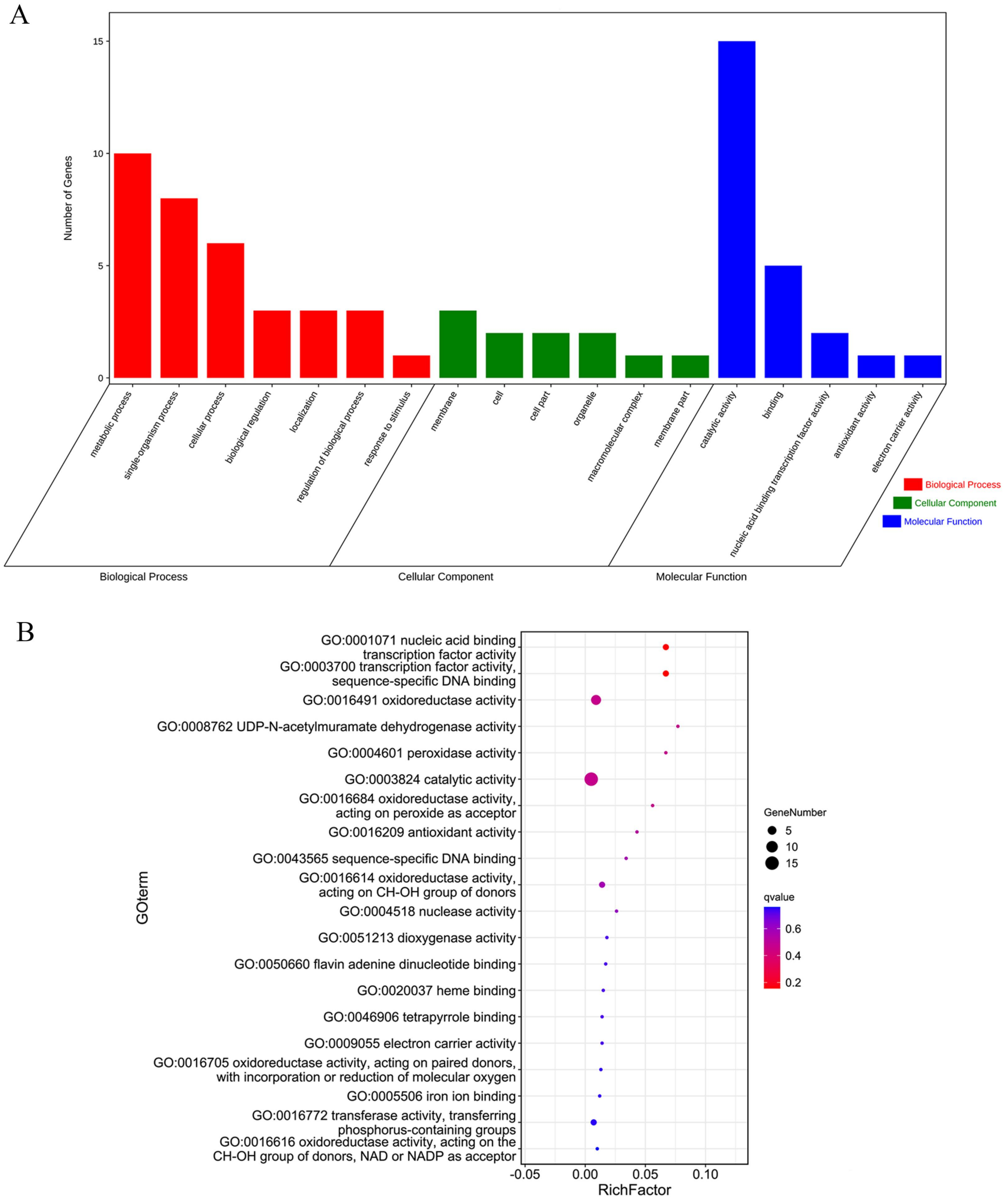


**FIG S5** GO and KEGG enrichment pathway of 487 specific proteins of *N.dimidiatum*.

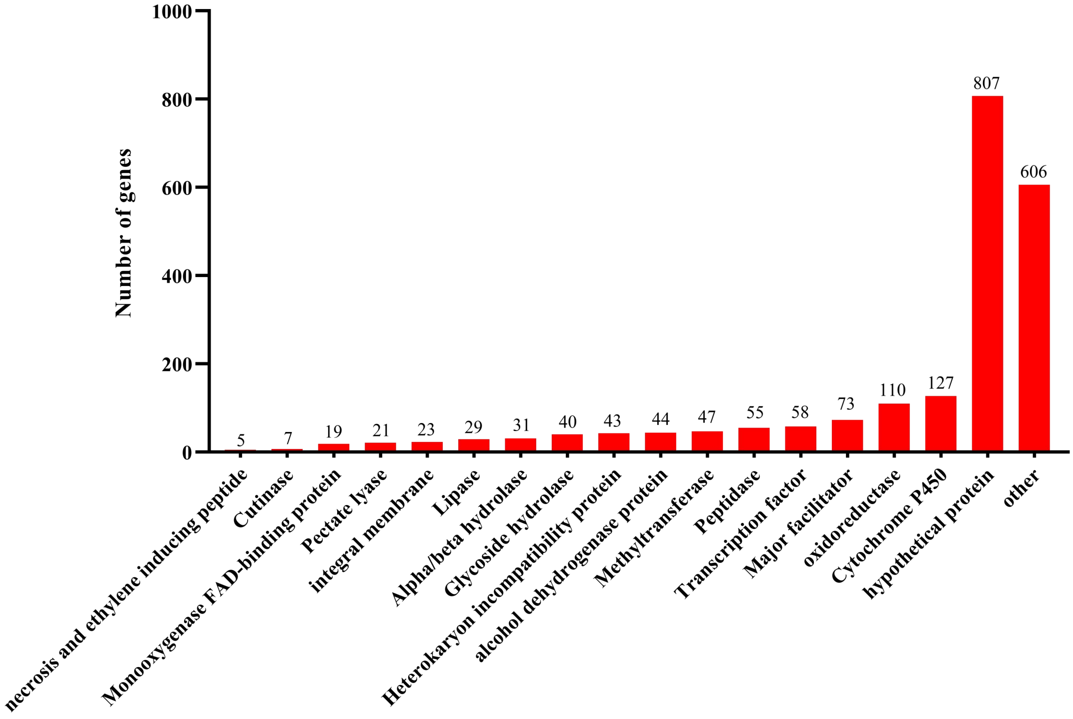


**FIG S6** Annotated statistical chart of 577 extended gene families of *N.dimidiatum*.

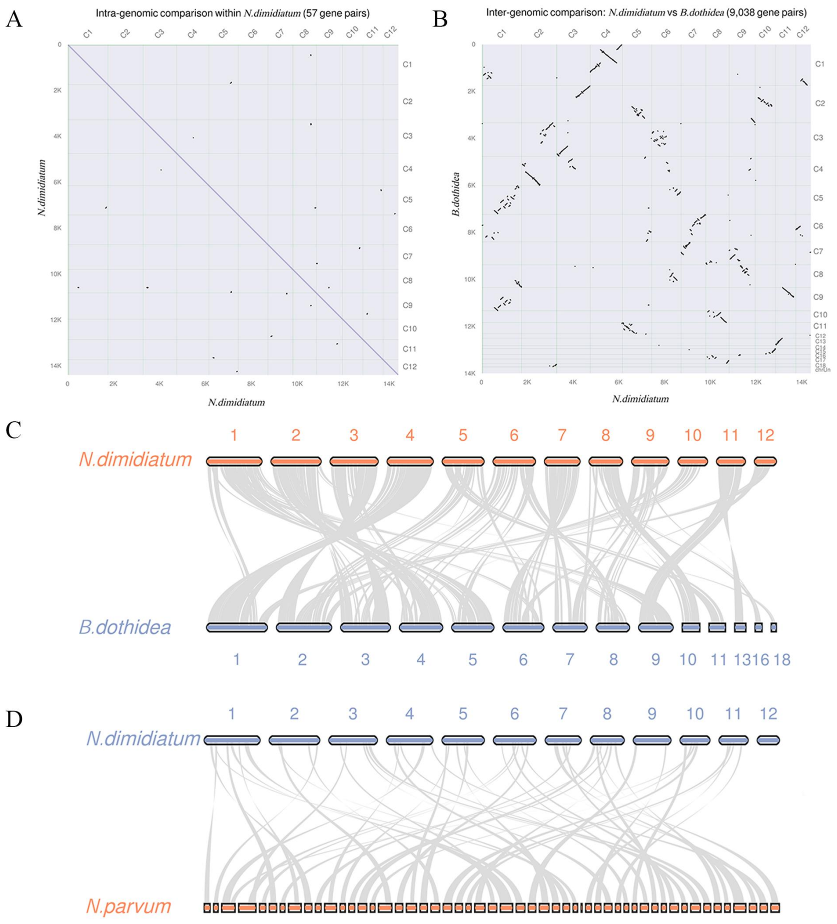


**FIG S7** Gene Syntenic Analysis. (A) Syntenic analysis of *N. dimidiatum* itself. (B) The distribution of orthologous genes between *N.dimidiatum* (x-axis) and *B.dothidea* (y-axis) chromosomes is consistent with the result of (C). (D) The distribution of orthologous genes between *N.dimidiatum* (x axis) and *N.parvum* (y axis) chromosomes.

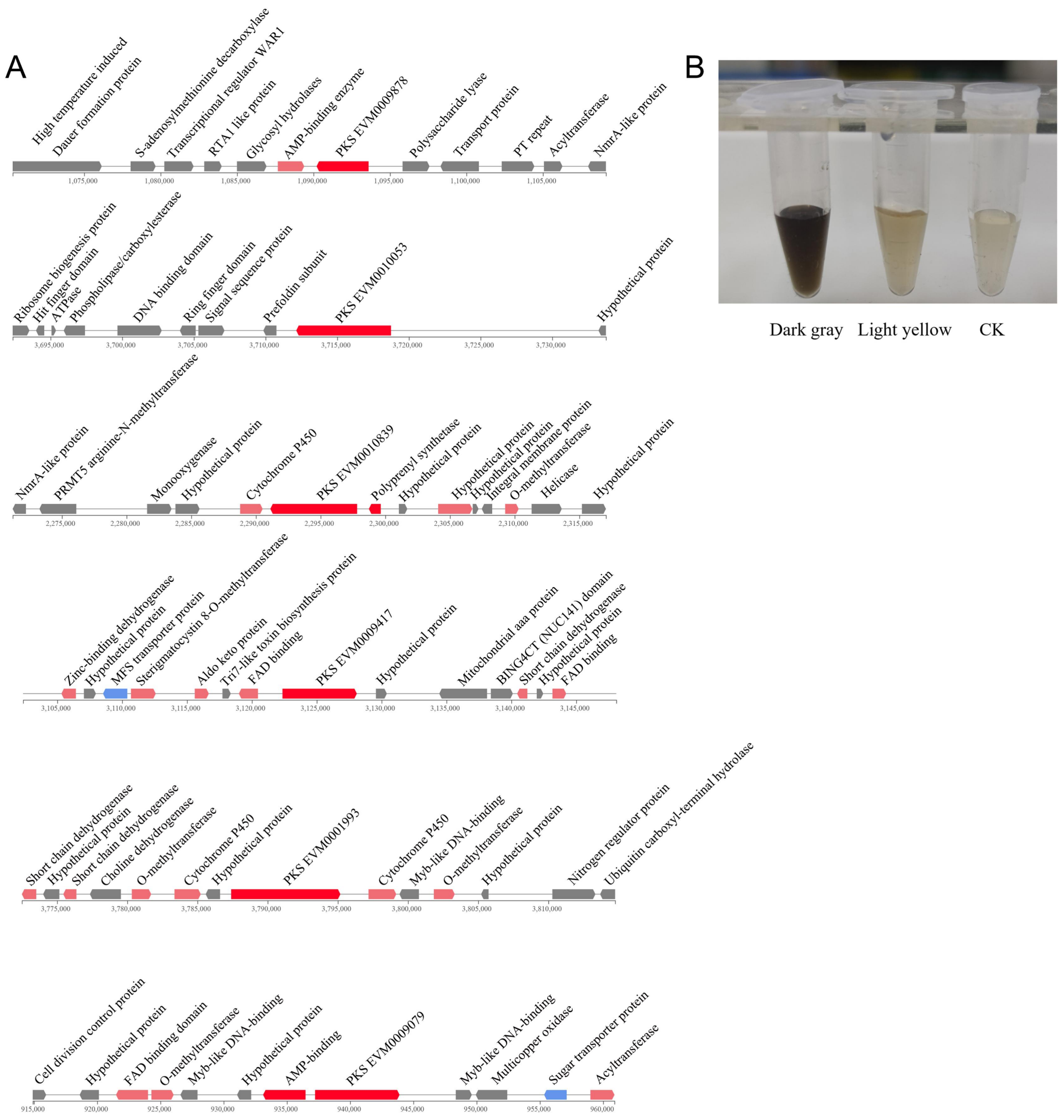


**FIG S8** (A) Six putative PKS gene clusters for pigment production in *N. dimidiatum*. Genes encoding transcription factors (e.g., *WAR1*), acyltransferases, cytochrome P450 enzymes, methyltransferases, MFS transporters, and o-methyltransferase were clustered with the PKS genes. These clusters are responsible for pigment synthesis. The bright red arrows indicate PKS genes. The dull red arrows indicate additional biosynthetic genes. The blue arrows indicate transport-related genes, and grey arrows indicate other genes. (B) Pigments produced by *N. dimidiatum* in supernatants that were collected at 4 days (light yellow) and 7 days (dark gray) after the mycelia

were subjected to starvation. Potato dextrose agar (PDA) medium served as the negative control (CK). Results are based on three biological replicates.

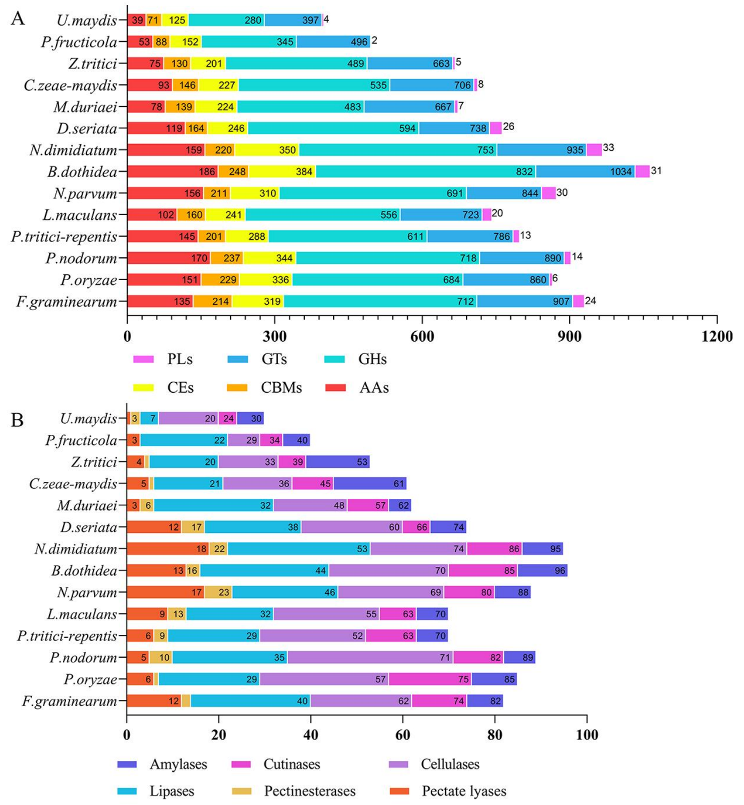


**FIG S9** (A) Numbers of putative carbohydrate-active enzymes (CAZymes) in 14 species. (B) Numbers of putative plant cell wall-degrading enzymes (PCWDEs) in 14 species. GHs: glycoside hydrolases, GTs: glycosyltransferases, PLs: polysaccharide lyases, CEs: carbohydrate esterases, AAs: auxiliary activities.

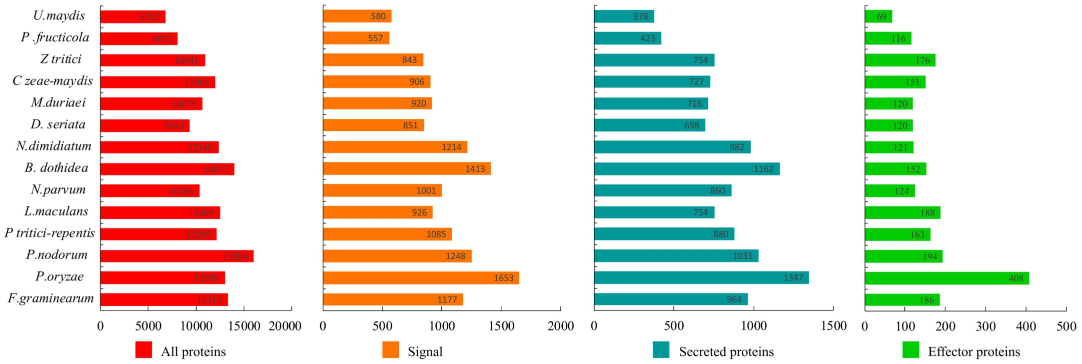


**FIG S10** Numbers of predicted genes encoding proteins, proteins containing signal peptides, secretory proteins, and effector proteins in 14 fungal pathogens.

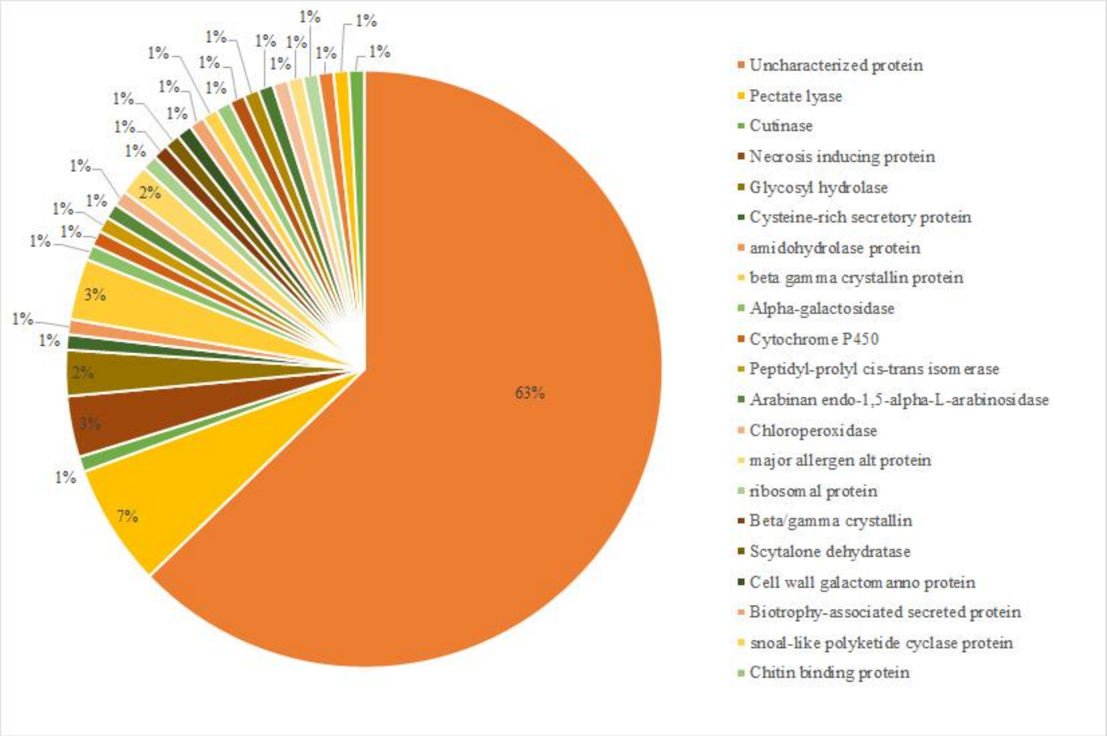


**FIG S11** 121 effector protein annotation information of *N.dimidiatum.*

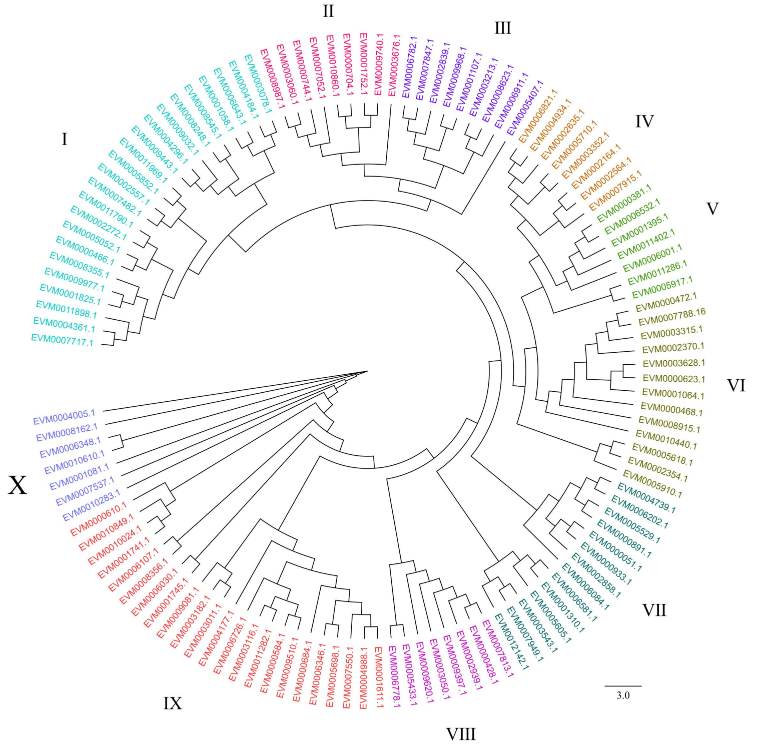


**FIG S12** Functional analysis of the effector of the presumed *N. dimidiatum*. The phylogenetic trees of 121 putative effectors were classified into ten super branches.

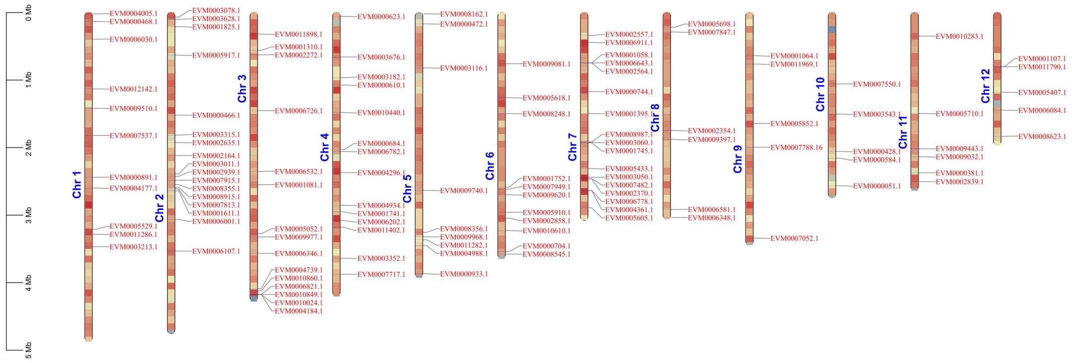


**FIG S13** The locus of 121 effector proteins on the chromosome of *N. dimidiatum*.
